# A Pilot Study of Increasing Nonpurposeful Movement Breaks at Work as a Means of Reducing Prolonged Sitting

**DOI:** 10.1155/2013/128376

**Published:** 2013-04-03

**Authors:** Dean Cooley, Scott Pedersen

**Affiliations:** University of Tasmania, Faculty of Education, Launceston, TAS 7248, Australia

## Abstract

There is a plethora of workplace physical activity interventions designed to increase purposeful movement, yet few are designed to alleviate prolonged occupational sitting time. A pilot study was conducted to test the feasibility of a workplace e-health intervention based on a passive approach to increase nonpurposeful movement as a means of reducing sitting time. The study was trialled in a professional workplace with forty-six participants (33 females and 13 males) for a period of twenty-six weeks. Participants in the first thirteen weeks received a passive prompt every 45 minutes on their computer screen reminding them to stand and engage in nonpurposeful activity throughout their workday. After thirteen weeks, the prompt was disabled, and participants were then free to voluntary engage the software. Results demonstrated that when employees were exposed to a passive prompt, as opposed to an active prompt, they were five times more likely to fully adhere to completing a movement break every hour of the workday. Based on this pilot study, we suggest that the notion that people are willing to participate in a coercive workplace e-health intervention is promising, and there is a need for further investigation.

## 1. Introduction

Increasing purposeful, or voluntary, physical activity both during leisure and work time is advocated as a means of reducing the risk of cardiovascular disease (CVD) [1–3]. In response workplace physical activity interventions have been designed to increase participation in purposeful physical activity programs during scheduled breaks in work time [4]. Yet the effectiveness of such interventions is mixed because of problems with sustainability and adherence [5]. Moreover, it appears that an increase in purposeful physical activity does not alleviate the CVD risk associated with prolonged periods of sitting (>4 hrs) [6–11]. Changes to the built environment, such as increases in technology, have resulted in prolonged occupational sitting time in excess of 6 hours [12], with concomitant decreases in energy expenditure for desk-based workers (>300 calories/per/day) [13, 14]. Guidelines for increasing cardiorespiratory fitness have changed over the years to reflect a growing understanding of the role of dose and frequency [15]. Recent evidence suggests short bouts of physical activity both purposeful and nonpurposeful (i.e., chores, standing up) are positively associated with cardiorespiratory fitness [15] and may buffer against issues of adherence to workplace health and wellbeing programs [4]. Moreover increasing nonpurposeful activity as part of an intervention may also ameliorate the health risks posed by prolonged sitting.

Research shows that short bursts of physical activity (<10 minutes) result in a reduction of CVD risk factors [16, 17]. For example, a cross-sectional analysis of the Framingham Heart Study Third Generation participants showed that accruing physical activity bouts of <10 minutes resulted in favourable changes to CVD risk [17]. There is evidence of the feasibility of these results being transferred to workplace interventions. For example, successful interventions may include a 10-minute flexibility and strength program [18], a single 10-minute bout of physical activity [19], mixed program for 30 minutes [20], or group physical activity classes [4]. Moreover, it appears that sustainability and adherence rates may be superior to those reported for purpose-based exercise interventions. In one study, average monthly attendance ranged from 76 to 86 percent over six months [4]. Given these results it would appear that short bursts of nonpurposeful activity may be suitable for worksites interventions. 

Purposeful activities are effective in improving cardiorespiratory fitness, but increases may not ameliorate the risks associated with prolonged sitting because the activities only occur once a day [21], and the seated position needs to be regularly interrupted throughout the day. A number of studies have shown that regularly breaking a seated position results in positive changes to glucose and lipid profiles [16], lipoprotein lipase [22], and metabolic risk [23]. Consequently, there is support for an increase in nonpurposeful movement into desk-based work [14, 24, 25]. One strategy for increasing nonpurposeful activity is changing the built environment by including ergonomic equipment that affords movement [13, 14]. In one study [26] middle-aged men who were either overweight or obese participated in three separate daily sitting schedules with a break of six days between each of the days. In the first trial condition, each participant sat for five hours with no break. In the second experiment, they walked on a treadmill desk at a light-intensity pace for two minutes every 20 minutes, and in the third trial condition they walked on a treadmill desk at moderate-intensity pace for 2 minutes every 20 minutes. Results revealed that light-intensity movement, such as standing up and moving regularly throughout the workday yielded health benefits. Nonetheless, despite promising results there is little data to suggest that installation of treadmill desks will result in people changing their prolonged sitting periods because their use reflects planned behaviour, and subsequently such a strategy may suffer from the same problems as other purposive movement interventions. Moreover, sitting for desk-based work reflects a habit rather than a planned behaviour, and therefore interventions may be better informed by theory of habits [27].

Sitting for desk-based work is a habit because it is a learned act that is automatically performed in the presence of situational cues [27, 28]. Initially, the decision to sit at work is likely to be under the control of constructs as described by the theory of planned behaviour [29]. Within the workplace employees are faced with a limited choice of built environment (i.e., chairs and fixed height desks), prevailing workplace attitudes towards what is required to complete the daily tasks, and social pressures to sit while working. These factors are likely to create a perception that one has little control to execute an alternative to sitting [30]. Nonetheless, developing a new habit is more complex than substituting a new behaviour for the existing behaviour, especially if the behaviour is complex such as those that require multiple decisions to be made in short periods of time.

Sitting for desk-based workers is a complex behaviour because it typically involves more than one action. For example, the worker has to turn on the computer, select an appropriate chair, acquire the necessary equipment for a job task on the desk, complete multiple tasks within short time frames, and decide upon the sequence of these tasks. The sequence of behaviours could be described as a habitual pattern [31], but it may contain semiautomatic responses [32, 33] or behavioural scripts [34], which require some level of conscious thought. Yet preexisting habits may cause a breakdown of the intention-behaviour relationship by overriding the intention to perform an alternative behaviour [27]. When an individual intends to perform a new behaviour but engages in an old behaviour it is described as a slip or action switch and is evidence of a strong habit intrusion [35]. The performance of counter-habitual goal-directed actions (e.g., standing while taking a telephone call instead of sitting) requires conscious attention to interrupt the habit. If attentional resources are absorbed by other tasks and the intention to perform an alternative course of action is not capable of “over-ruling” the activation of a habitual programme [35–37], an action slip may result. For example, a desk-based worker under a high stress load because of attending to multiple tasks in a short time frame may find that when the phone rings, rather than stand as they have been instructed to do to break prolonged occupational sitting time (alternative course of action) they find themselves maintaining the old habit of sitting. The issue for interventions designed to break prolonged sitting is how to increase the odds of people performing an alternative behaviour. 

One mechanism for counteracting the effect of an existing habit on a new habit is through the use of prompts at the point of decision [27, 28, 38]. Prompts at points of decision include a wide range of mechanisms including signs, emails, text messages, or telephone calls [39]. The rationale is the prompt presents a situation whereby the individual is able to reevaluate behavioural choices [38]. There is research that supports the efficacy of prompts to help people decide to participate in alternative health behaviour. These changes typically involve the use of electronic prompts and reminders to encourage people to engage in a particular behaviour [40]. For example, one study showed that a simple message at the point of decision extoling the health benefits of taking the stairs over the escalator resulted in an increase in stair use over baseline measures [38]. In terms of the efficacy of prompting to change prolonged sitting one study [41] showed that participants who received a computer-based prompt significantly reduced their sitting times compared to their counterparts who received education only about the health effects associated with prolonged sitting. Nonetheless, the effect was observed only over a 5-day period within a clinically controlled environment.

Despite the promising results for interventions to reduce prolonged sitting thus far, there are some limitations to consider. Changes to the built environment, such as having workers use treadmill desks, may result in an increase in work errors [42]. Second, the assessment of efficacy of ergonomic posture equipment is restricted to clinical studies, with no data available from field-based research to provide an understanding of how such changes to the built environment might work or be implemented in a workplace. Thus the applicability and sustainability of such equipment in workplaces to break prolonged sitting is unknown. Moreover, the financial costs associated with ergonomic workplace equipment may be prohibitive to small organisations. Finally, the efficacy of prompts is also mixed, with numerous variables influencing their effectiveness [39, 43]. Further, no field studies have reported the sustainability of such an intervention to break prolonged sitting in the workplace. 

Although prompts at points of decision alert individuals about alternative behaviours, the individual can either consciously or unconsciously (i.e., action slip) ignore the prompt. This is a common occurrence in other health habits such as seat-belt wearing [44] and cell phone use while driving a car [45]. Thus, strategies should go beyond the usual range of education, rehabilitation, punishment, reward, and disincentive schemes to change individual attitude and belief about health behaviour [32] and environmental modifications to achieve significant behavioural changes among the target population [46]. We believe that it is timely to revisit some of the notions within a communitarian model of health promotion [47–49], in particular the active-passive approach for individual behaviour and preventative health interventions. Within this model when individuals voluntarily performing a preventative health action (e.g., regularly breaking sitting posture during the workday) it is considered an active prevention strategy. Thus, only individuals who choose to engage in the behaviour would gain the protective health benefit associated with that of regular standing, whereas passive prevention strategies remove some or all of the decision making process from the individual (e.g., only having water available in the drink machines). To assess this model we utilised a workplace e-health program that allowed us to manipulate the delivery of passive and active prompts in an effort to have desk-based employees engage in nonpurposeful movements throughout the workday as a means of reducing their prolonged occupational sitting time.

For health interventions there are a number of benefits in adopting a more passive approach. Health interventions that are passive have higher compliance levels and therefore greater efficiencies [44, 49–53]. Of note is the use of persuasive systems, which are computerised software systems designed to change or shape attitudes or behaviours [52, 53]. Noncoercive [52] and coercive [53] persuasive systems have been successful in changing health behaviours. Given the less than successful outcomes of purposeful physical activity interventions in the workplace which have relied on an active approach to preventive health [5], we hoped to learn if desk-based employees would tolerate a coercive, passive-prompting e-health intervention. We were also interested in differences between passive-based and active-based prompts for maintaining adherence to the health behaviour designed to increase nonpurposeful movement throughout the workday. We hypothesised that a passive prompt, compared to an active prompt, would significantly increase the odds of participants complying with seven periods of non-purposive movement during a workday. Noncompliance to the prompting conditions was defined as recording between one and six activities per workday. This number was based on the maximum number of prompts that a participant could receive during an eight-hour shift with a one-hour lunch break. 

## 2. Methods

### 2.1. Sample

Participants were randomly selected from approximately 460 desk-based employees to take part in a field-based, sequential intervention study within a professional workplace across multiple sites (Project PAUSE). Given the lack of previous studies to guide power analysis and the pilot nature of this study, we chose a relative wide precision estimate of 28% with 95% confidence intervals, which indicated that a sample size of 50 was needed. Participants were randomly recruited through a computerised random draw. Eligibility criteria for inclusion into the study were (a) full-time desk-based employment, (b) clear of any medical health issues, and (c) daily desktop computer access to the internet. 

In terms of the sample frame, the organisation's full-time desk-based workers were largely composed of females (67%); so, for parsimony, we chose a 1/3 split for our sample. Four participants withdrew from the study because of personal reasons two days before the induction and were not replaced, as replacements could not attend the induction at short notice. So the study proceeded with a reduced sample (*N* = 46). Participants had a range of work roles including receptionists, forensic analysis, administrative support, call center, sworn duties, and media/community liaison. Approximately 80 percent of participants worked in urban-based offices. Workplace configurations included open plan worksites, single offices, and shared office spaces. All participants completed the Exercise Stages of Change questionnaire [54], which assesses individual motivation to participate in exercise. Participants (females = 33, male = 13) all reported being in one of the first three stages of exercise participation (i.e., precontemplation (23%), contemplation (58%), or preparation (19%)), with no participant indicating that they had started or had been involved in a regular exercise program for the previous six months. Before data collection participants provided informed consent in accordance with granted university ethics committee procedures (H10875). Demographic characteristics of the sample are presented in Table 1.

### 2.2. Procedures

All participants first attended an induction session to inform them about the protocol of the study. The sequence of the session was the collection of demographic baseline data (30 minutes), an educational session on the negative health effects associated with prolonged sitting (15 minutes), general instructions on the recommended dose of movement to alleviate the adverse effects of prolonged sitting (20 minutes), and an informational session on using the e-health software (30 minutes). Participants were instructed that the length of the study would be 26 weeks and were reminded that their involvement in the study was strictly voluntary and they could withdraw at any time. At the completion of this induction session, all participants had the e-health software installed onto their computers. Participants were not blinded to other participants' involvement because of the nature of some worksites, and all attended the one induction session. 

### 2.3. Intervention

The intervention was an e-health software program designed to passively prompt employees to break prolonged sitting periods by increasing nonpurposeful workday movement. The software has two distinct phases. It contains a set timed prompt that reminds employees to break their sitting time by engaging in nonpurposeful movement throughout the workday. The software provides employees a choice of 60 office-appropriate activities (i.e., walking, taking the stairs, and retrieving the photocopies). As the aim of the intervention was to increase nonpurposeful movement during the workday, participants were free to choose the activity, duration, and intensity. This e-health program was tested through passive and active prompting strategies.

### 2.4. Passive Prompt Condition

**Figure 1:**
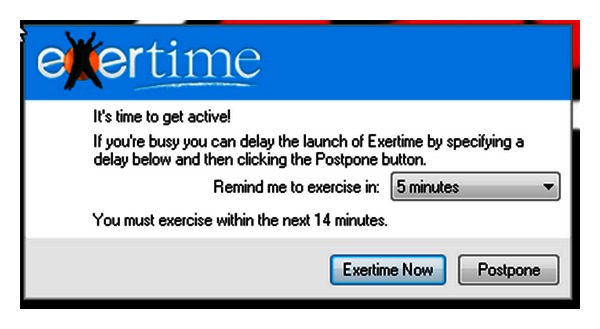
Prompting message seen by participants on their computer screens.

The passive prompting condition was initiated during the first 13 weeks of the study. During this period, participants were exposed to a prompt that did not allow them to ignore the request to engage in the recommended behaviour. This was achieved through two mechanisms that were occurred in the following sequence. First, a small icon appeared on the taskbar. After 45 minutes the small icon automatically enlarged into a coloured pop-up prompt (5 cm × 3 cm) onto the bottom right hand side of participants' screens. The prompt contained a message alerting participants that the movement sequence was about to start (Figure 1). 

Second, after the prompt appeared, a countdown clock started, and after 60 seconds, participants' screens were de-activated, and a cover screen appeared revealing the e-health software interface. At this point of decision, participants could select to complete a movement activity. Participants had freedom of choice over which activity to perform and the frequency or duration of participation. When participants completed their chosen activities, they were then prompted to record their progress. These activities were time stamped and stored on a remote server so that we were able to calculate the total number of logged activities for each day. Logged activities served as our dependent variable of compliance. Once data were recorded, the sequence terminated, and the participants regained access to their original computer screen at the point of deactivation. The prompt would then reinitiate after 45 minutes.

The dependent variable was determined by calculating the total number of days with seven or more logged activities and the total number of days with one to six logged activities. We set the criteria for compliance at seven or more activities per day because this indicated that participants were regularly breaking their prolonged sitting every hour as indicated by the Australian national guidelines [55]. We excluded days where no activity was logged because this may have reflected that the participant was out of their office, on leave, or working at other worksites with no computer. As this was a self-report measure, participants received a phone call from the researchers once throughout the study period to remind participants about the necessity to accurately report their activities. 

### 2.5. Active Prompted Condition

After 13 weeks of the passive prompting condition, the researchers disabled the timed prompt for a further 13-week period. During this phase, if the participants wanted to engage with the e-health software, they needed to do so under their own volition. Participants could still view the icon on their taskbar and could still use the e-health software but had to voluntarily initiate the program by clicking on the icon. Once voluntarily initiated, the sequence remained the same as that for the passive prompting condition, and the dependent variable of logged activity frequency to determine compliance was used in the same manner. 

### 2.6. Data Analysis

Using commercially available software [56] odds within conditions (compliance/noncompliance), odds ratio (OR), and 95 percent confidence intervals for the OR were generated using a 2 (compliance/noncompliance) × 2 (passive prompt/active prompt) contingency table. For each prompt condition, total number of days compliant and noncompliant for the 26-week experimental period were calculated and used as the frequency measure. A test of the hypothesis (OR = 1) was assessed using a chi-square statistic.

## 3. Results

All participants (*N* = 46) maintained the software on their desktop computers for the 26 weeks of the study and recorded activities through the study. Participants recorded a total of 2893 days out of a possible 5980 days where at least one activity was logged for the day across the 26-week study period. Participants recorded using a wide variety of movement-based activities, with the most popular activities being stair climbing, walking, and chair squats. Most days of recorded activity (Table 2) were associated with the passive prompted condition (*n* = 2321 days). Similarly, the highest number of activities recorded for a day by an individual was 23 in the passive prompt condition compared to 16 activities for the active prompt condition. For the passive prompt condition, nine activities in a day were the most frequent count (12%) compared to one logged activity per day for the active condition (37%). 

In terms of each prompting condition, results showed that odds of compliance were greater in the passive condition (Odds = 1.1) compared to the active condition (Odds = 0.23). In terms of comparing the two conditions, a passive prompt improved the odds of desk-based workers complying to participate in nonpurposeful movement every hour seven times a day nearly five times more compared to the active condition (OR = 4.78, 95%CI = 3.78–5.93, and *P* < 0.05). Therefore, participants who were automatically reminded and forced to make a decision about moving were significantly more likely to engage in nonpurposeful physical activity seven times a day compared to when they were left to spontaneously stand throughout the workday on their own accord. 

## 4. Discussion

The first finding of this study was that the employees in our sample were willing to accept a passive-based workplace e-health intervention [48] that was predicated on the principles of introducing nonpurposeful movement. No participant withdrew from the study during the 26-week study, although not all participants achieved our rigid criteria of full compliance by completing seven nonpurposeful movement breaks throughout the workday. Given that CVD is a major health issue [1–3] and the widening of guidelines for physical activity dose and frequency [15], these results are encouraging. Adherence to purposeful physical activity intervention are a constant problem. One cause of low adherence and compliance rates is some recruits to purposeful-based physical activity interventions cannot meet the high dose and frequency rates set for increases in cardiorespiratory fitness because of a variety of factors. Our results give an indication that people who do not exercise regularly and are willing and capable of increasing nonpurposeful movement activities that were associated with their work if regularly prompted to do so. Given the number of people who are not regularly participating in purposeful physical activity [5], CVD risk could be somewhat addressed through workplace physical activity interventions based on increasing compulsory nonpurposeful movement.

Our second finding was the enhanced benefit of using a passive prompt compared to an active prompt to achieve increase adherence to new health behaviour. A workplace e-health intervention underpinned by a passive prompt increased compliance by nearly five times to increasing nonpurposeful movement during the workday compared to when employees were left to their own free will to comply with the intervention. Within the context of passive versus active approaches to interventions [48] we believe this is the first field-based report of desk-based employees accepting a coercive persuasive system within a preventative health intervention designed to increase nonpurposeful movement at work. Given the low compliance rates for the active condition, which occurred after the passive condition for all participants, it could be assumed that the new behaviour of participating in hourly nonpurposeful movement had not become habitual. Despite the best intentions of health professionals, interventions designed to increase purposeful activity (e.g., walking, running, and strength training) have returned mixed results [5]. Our results provide preliminary evidence for the use of coercive persuasive systems to ameliorate low compliance rates may work with other health preventative initiatives. 

Finally, the compliance results in the active condition, which followed the passive condition for all participants in our sample, highlight issues with sustainability of workplace physical activity-based interventions. The difficulty health professionals have in establishing new behaviours is well documented in the literature [57]. It would appear that 13 weeks of exposure to a coercive prompt is insufficient time to establish the nonpurposeful movement as a new behaviour. Sitting at work is a complex behaviour that involves many subroutines, and thus longer periods of exposure to the passive prompt may be needed to develop the new habit of regularly breaking sitting posture to move. Or as others have [58] argued, an insistent and obtrusive reminder might well be effective in the short run, but the effect reduces significantly over time. We would advocate that future research investigates a multiple strategy approach for changing workplace health behaviours to include changes to the built environment, such as treadmill or standing desks, coupled with a passive prompt to encourage workers to become less sedentary during the workday. 

Our results have several possible implications. Coercing people to comply with health recommendations has the potential to reduce wastage of personal and monetary resources, help improve the efficacy of health and wellbeing interventions, and, most importantly, potentially reduce mortality and morbidity associated with preventable diseases such as CVD. Yet, the palatability of using passive approaches for individual preventative health interventions, such as increasing physical activity levels, has yet to be fully tested by researchers. Further, to date, despite the widespread knowledge of the effect of base levels of exercise (e.g., walking 30 minutes per day) on health and wellbeing, health researchers lament the low adherence rates within the population to recommended dose levels yet remain largely silent and inactive in recommending and prescribing nonpurposeful movement as an alternative or an adjunct strategy.

Nonetheless, there were several limitations to this study. This was a pilot study to test the feasibility of the e-health software and the efficacy of applying a passive persuasive system to a workplace e-health intervention. We did not measure habit strength and thus are unable to determine if participants' nonpurposeful movement habits had fully developed by the end of passive prompt condition. Hence, the poor compliance rates during the active condition may reflect that the behaviour had not become habitual. Second, in the active condition, the small number of reports for both compliance and noncompliance may lead to an under-estimation and hence alter the final odds ratio calculation. Third, we are unable to establish if participants may have chosen to simply stand to break their sitting time and elected not to complete a period of nonpurposeful activity in either condition. Finally, the measure of compliance was based on self-report. It is possible that participants simply became annoyed with the prompts or software and chose to record a false activity or no activity because of time and work pressure. Follow-up studies should incorporate a larger sample size and use more direct measures of compliance, such as accelerometers, in conjunction with e-health, passive-prompting software programs. Moreover, there is scope to determine the saliency of the coercive approach with other health behaviours. 

## 5. Conclusion

Results from this study found that desk-based workers who received information about the health effects of prolonged sitting and who subsequently participated in a workplace e-health intervention based on a passive approach model had significantly higher compliance levels to participate in nonpurposeful movement compared to voluntary engagement in the same program. Further research is needed to rigorously test the efficacy of a such an approach in terms of sustainability, as well as what conditions employees are willing to be coerced into changing their health behaviours while at work. We plan to conduct further studies on a larger sample of desk-based workers across multiple worksites to help understand how health can be positively influenced within the workplace in an effort to reduce the risks associated with CVD. 

## Figures and Tables

**Table 1:** Demographic characteristics of the sample. Values are means standard deviations.

Variable	Before	After
Age (years)		
Female	41.53 (12.1)	
Male	46.10 (6.3)	
Height (cm)		
Female	164.61 (6.74)	
Male	176.76 (6.85)	
Weight (kg)		
Female	71.91 (13.45)	70.87 (11.88)
Male	96.31 (17.96)	95.46 (17.28)
BMI		
Female	26.55 (4.36)	26.19 (3.96)
Male	30.81 (5.17)	30.52 (4.90)

**Table 2:** Total number of days for compliance and noncompliance in each prompt condition.

	Passive prompt	Active prompt
Compliance	1216	108
Noncompliance	1104	465
